# Antegrade Irrigation via a Percutaneous Nephrostomy Tube During Rigid Ureteroscopic Laser Lithotripsy for Distal Ureteral Stones: A Case Report

**DOI:** 10.7759/cureus.113611

**Published:** 2026-07-29

**Authors:** Hasan R Al Shabaan, Nidhal Haddad, Mohammed A AlBesher, Abdulhakeem Almarzooq, Hussain M Albin Hamdhah

**Affiliations:** 1 Urology, King Fahad Hospital Hofuf, Al-Ahsa, SAU; 2 Urology, Prince Mohammed bin Abdulaziz Hospital, Madinah, SAU

**Keywords:** antegrade irrigation, distal ureteral stones, holmium laser lithotripsy, percutaneous nephrostomy tube, stone retropulsion, ureteroscopy (urs)

## Abstract

Stone retropulsion during ureteroscopy (URS) remains a significant challenge that can prolong operative time and may require additional procedures. Antegrade irrigation through a percutaneous nephrostomy (PCN) tube has been described primarily for proximal ureteral stones. We report the successful application of this technique in a patient with bilateral distal ureteral stones using pre-existing bilateral PCN tubes during rigid ureteroscopic laser lithotripsy.

A 50-year-old woman presented with bilateral flank pain, dysuria, hematuria, and fever. Laboratory findings showed elevated creatinine levels and leukocytosis. Non-contrast CT revealed multiple bilateral distal ureteral stones, the largest measuring 12.4 mm, with moderate-to-severe hydroureteronephrosis and left lower calyceal stones. Bilateral PCN tubes were inserted, resulting in clinical and biochemical improvement. Two weeks later, elective bilateral rigid URS with laser lithotripsy was performed under continuous gravity-assisted antegrade saline irrigation through the PCN tubes.

The antegrade flow promoted the distal migration of stone fragments, minimized proximal retropulsion, and maintained a clear visual field. All fragments were successfully retrieved using a Dormia basket, and bilateral double-J stents were placed. At four weeks, a postoperative kidney-ureter-bladder (KUB) radiograph showed no visible residual radiopaque stones. No irrigation-related complications were observed.

In conclusion, antegrade irrigation through PCN tubes may be a practical, low-cost adjunct that facilitates fragment management and may improve stone-free outcomes, even for distal ureteral stones, particularly in patients requiring initial urinary diversion. This technique warrants further evaluation in larger studies.

## Introduction

In patients with obstructing ureteral stones, particularly those experiencing persistent pain despite analgesic therapy or acute renal impairment, urinary diversion using percutaneous nephrostomy (PCN) or ureteral stenting may be necessary [1-4].

The most frequently used procedures for treating ureteral stones are ureteroscopy (URS) and extracorporeal shock wave lithotripsy (ESWL). ESWL has traditionally been used for proximal ureteral stones, whereas rigid ureteroscopic stone removal has been preferred for the surgical treatment of distal ureteral stones. However, URS for both proximal and distal ureteral stones can result in stone retropulsion, potentially prolonging operative time and necessitating additional procedures to remove any remaining stones [1,2].

Moreover, residual stone fragments may cause persistent renal colic, recurrent stone formation, and infection. The challenge may be greater when a rigid ureteroscope is used, as it may be unable to access the intrarenal collecting system. Numerous anti-retropulsion techniques, including the Stone Cone, N-Trap, and lubricating lidocaine jelly, have been used to prevent stone retropulsion [3].

Antegrade irrigation through a PCN tube has emerged as an alternative strategy for reducing proximal stone migration. By creating a continuous antegrade flow from the renal collecting system toward the bladder, this technique may improve endoscopic visualization, facilitate the distal migration of stone fragments, and reduce intrarenal pressure during URS. A previous study demonstrated encouraging outcomes with antegrade irrigation during semirigid URS for proximal ureteral stones, including higher stone-free rates and shorter operative times without increased complication rates [4].

Despite these promising results, the use of antegrade irrigation through pre-existing PCN tubes during semirigid URS for distal ureteral stones has rarely been described in the literature. Patients who undergo initial urinary diversion using PCN for obstructing ureteral calculi represent a unique opportunity to utilize the existing nephrostomy access as an adjunct during definitive treatment with URS.

Herein, we report the successful management of bilateral distal ureteral stones using a semirigid ureteroscope with continuous antegrade irrigation through pre-existing bilateral PCN tubes. We describe the surgical technique and discuss its potential advantages, limitations, and clinical applicability in selected patients.

## Case presentation

A 50-year-old woman with no significant past medical history presented to the ED with a two-week history of bilateral flank pain associated with dysuria, gross hematuria, and fever. On presentation, her vital signs included a pulse rate of 100 beats/min, blood pressure of 130/70 mmHg, and temperature of 38.0°C. Physical examination revealed a soft abdomen with bilateral flank tenderness.

Initial laboratory investigations demonstrated leukocytosis, with a white blood cell count of 13 × 10⁹/L, and an elevated serum creatinine level of 124 µmol/L. Urinalysis showed no evidence of UTI, while subsequent urine and blood cultures demonstrated no bacterial growth. Despite the negative microbiological findings, empirical IV antibiotics were initiated because of clinical suspicion of an obstructed upper UTI based on the patient’s fever, leukocytosis, bilateral urinary tract obstruction, and perinephric inflammatory changes on imaging. The laboratory findings are summarized in Table 1.

**Table 1:** Laboratory investigations at presentation and after bilateral percutaneous nephrostomy (PCN) tube insertion. PCN: Percutaneous nephrostomy; µmol/L: Micromoles per liter; ×10⁹/L: 10⁹ cells per liter.

Parameter	On presentation	After bilateral PCN tube insertion	Reference range/expected result
Serum creatinine	124 µmol/L	80 µmol/L	53-97 µmol/L
WBC count	13 × 10⁹/L	6.9 × 10⁹/L	4.0-11.0 × 10⁹/L
Urinalysis	No evidence of infection	Not reported	Negative for leukocytes, nitrites, and bacteria
Urine culture	No growth	Not reported	No growth
Blood culture	No growth	Not reported	No growth

**Figure 1:**
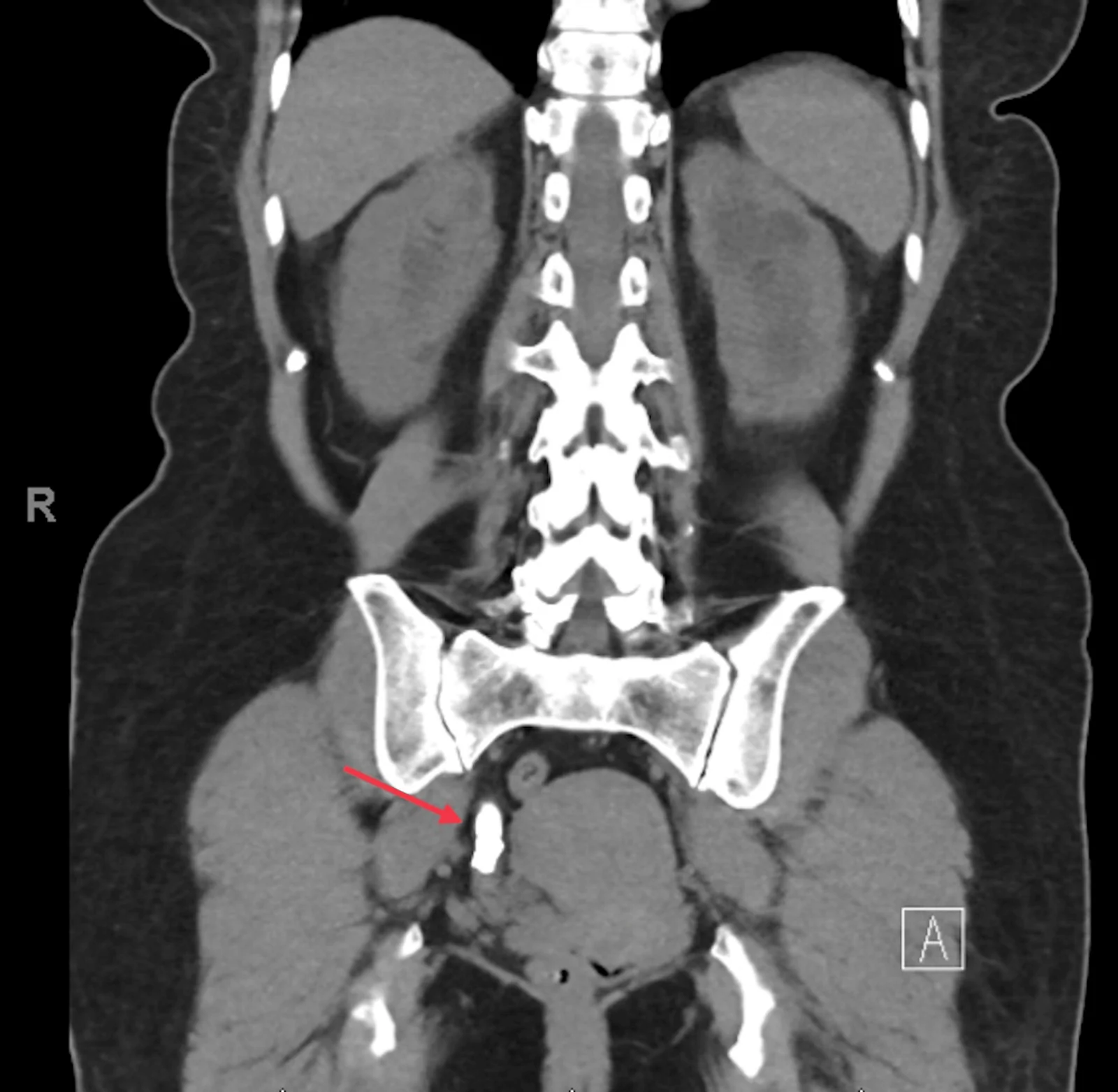
Coronal non-contrast CT image showing right distal ureteral stones. The red arrow indicates one of the stones.

**Figure 2:**
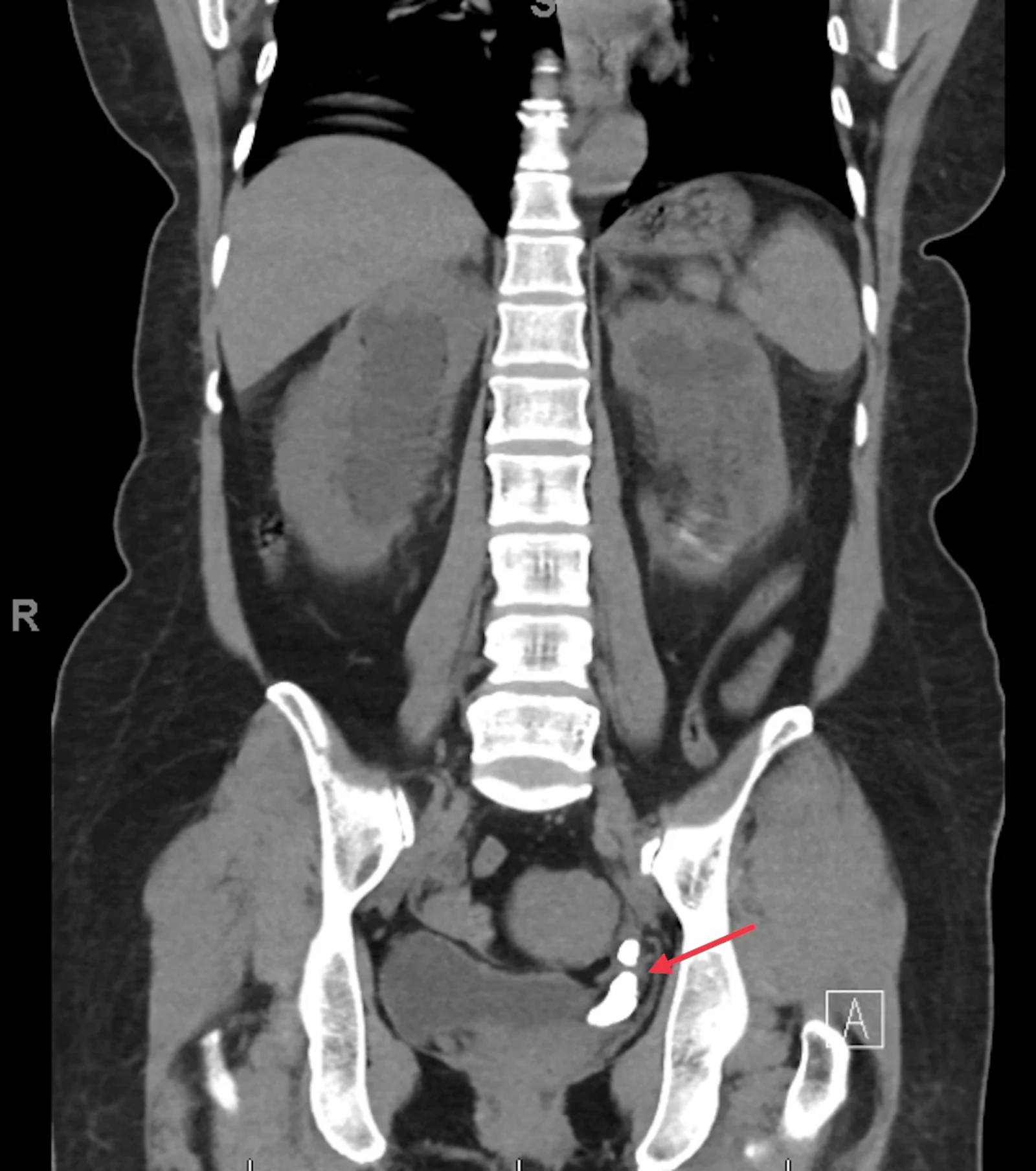
Coronal non-contrast CT image showing left distal ureteral stones. The red arrow points to the stone.

**Figure 3:**
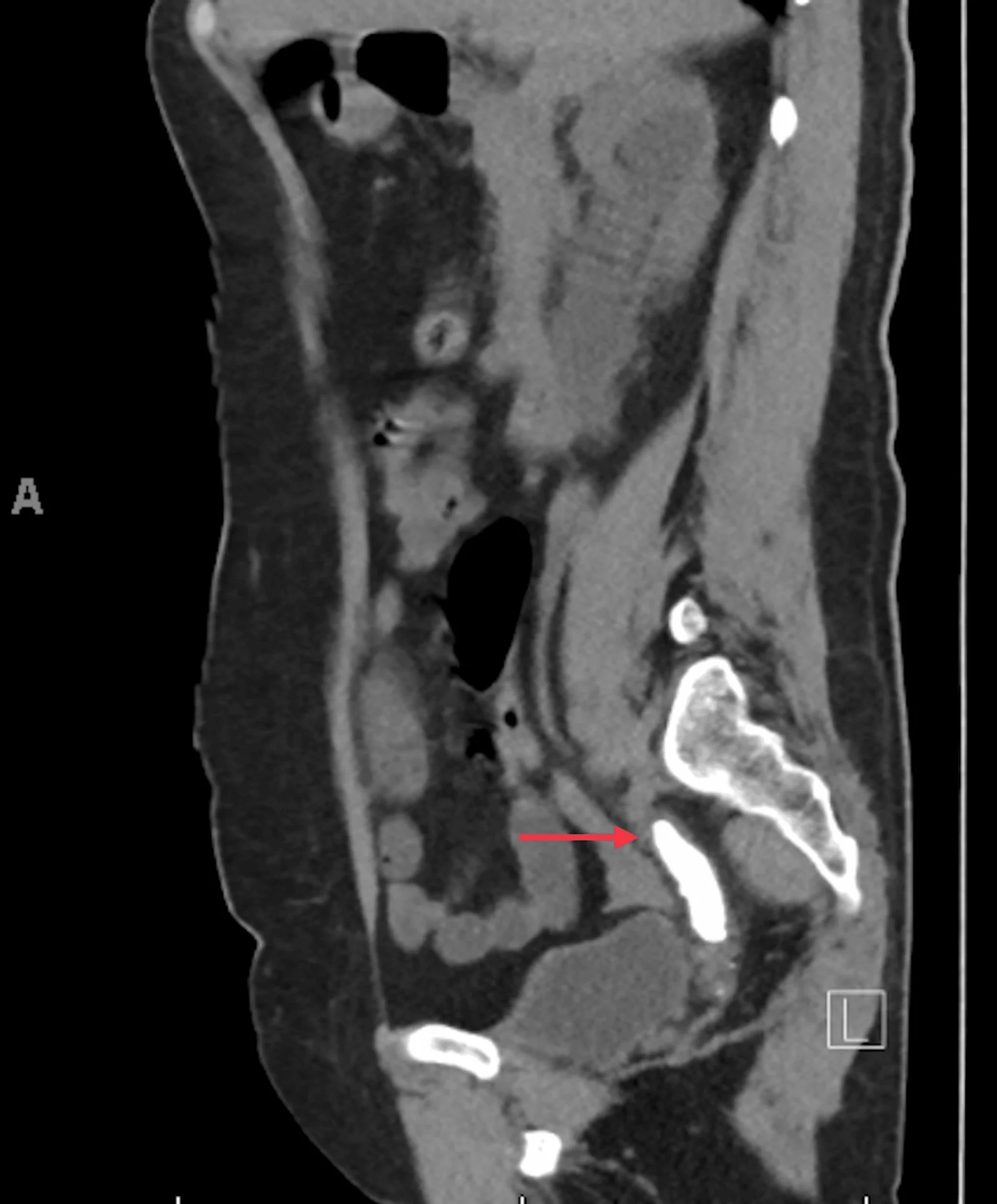
Sagittal non-contrast CT image showing right distal ureteral stones. The red arrow points to the stone.

**Figure 4:**
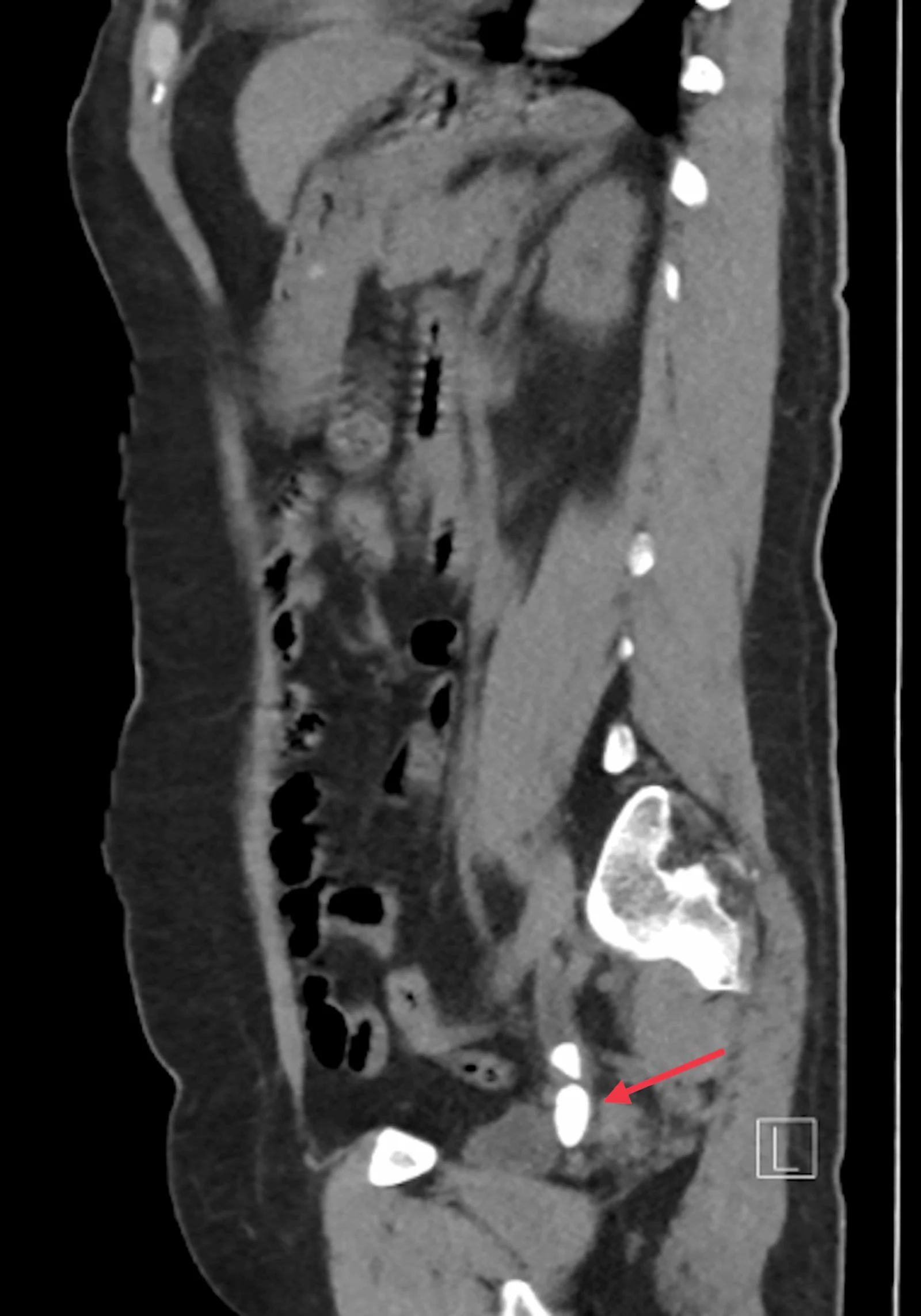
Sagittal non-contrast CT image showing left distal ureteral stones. The red arrow points to the stone.

Non-contrast computed tomography (NCCT) of the abdomen and pelvis demonstrated multiple bilateral distal ureteral calculi causing moderate-to-severe bilateral hydroureteronephrosis, with associated perinephric fat stranding (Figures 1-4). The largest left distal ureteral stone measured 12.0 × 10.4 mm, while the largest right distal ureteral stone measured 12.4 × 9.0 mm. The mean stone density was 550 Hounsfield units (HU).

**Figure 5:**
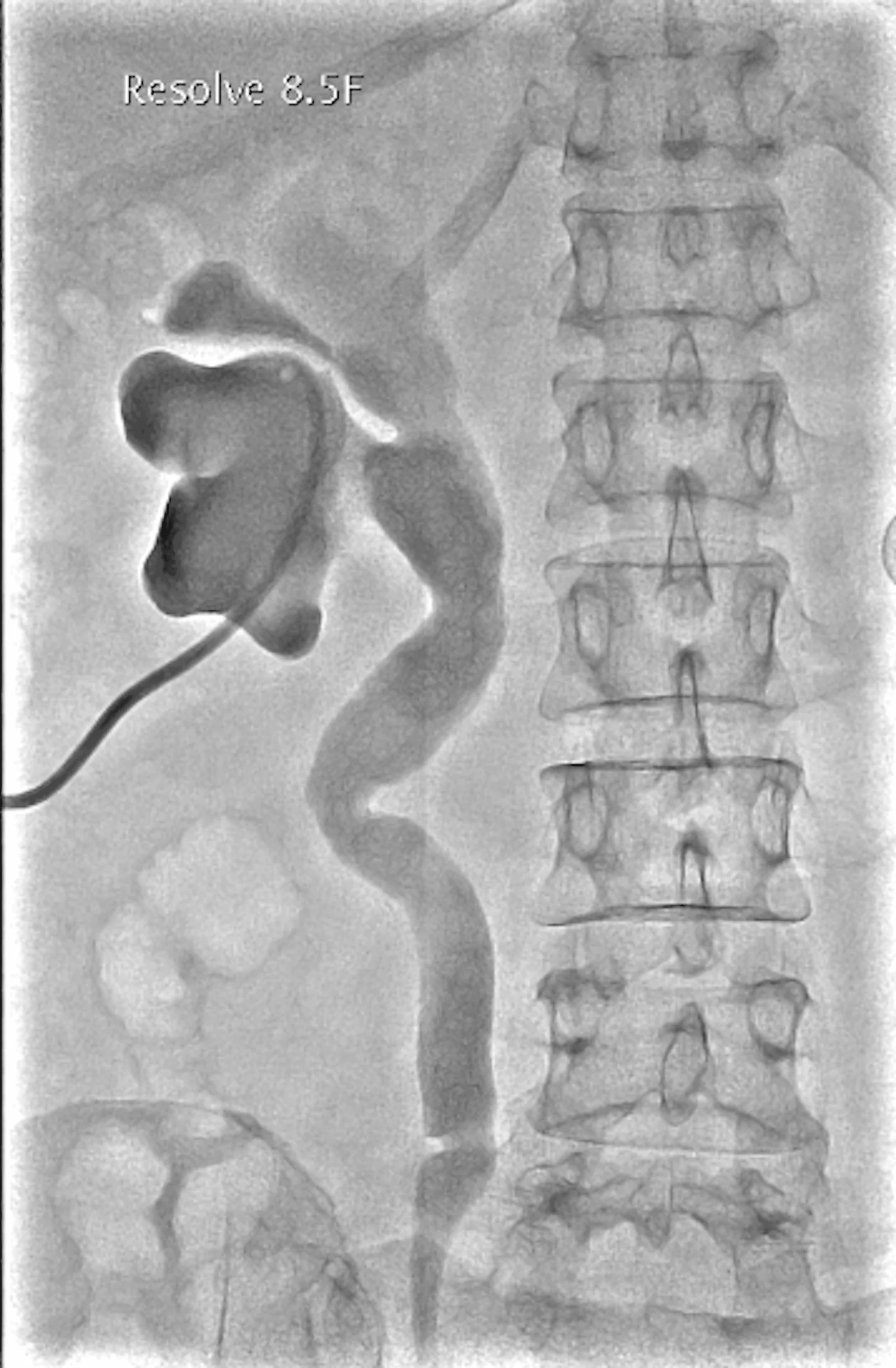
Nephrostogram obtained after insertion of the right nephrostomy tube showing a dilated pelvicalyceal system.

**Figure 6:**
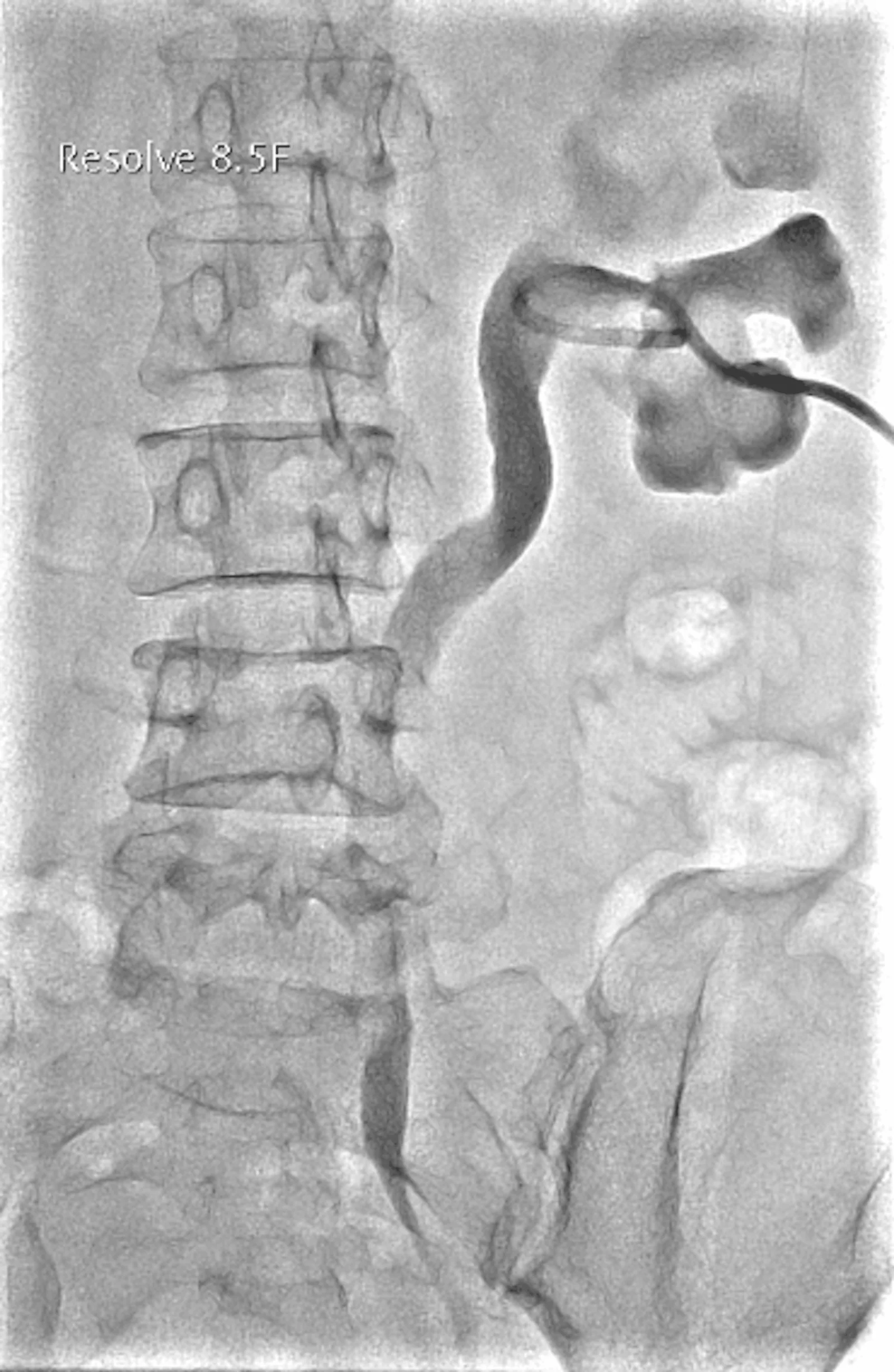
Nephrostogram obtained after insertion of the left nephrostomy tube showing a dilated pelvicalyceal system.

Given the presence of bilateral obstructing ureteral stones with impaired renal function and suspected infection, bilateral 8-Fr PCN tubes were inserted by the interventional radiology team to achieve urgent urinary decompression (Figures 5-6). Following drainage, the patient’s clinical condition improved, and her renal function normalized, with a serum creatinine level of 80 µmol/L. She was discharged after a three-day hospital admission.

Two weeks later, the patient was readmitted electively for definitive stone management. She underwent bilateral semirigid ureteroscopy using a 7.5-Fr ureteroscope under general anesthesia. Laser lithotripsy was performed using a holmium:yttrium-aluminum-garnet (Ho:YAG) laser with a 356-µm laser fiber. The laser settings were 0.6-1.0 J at 10-15 Hz. Continuous gravity-assisted antegrade irrigation was provided through the pre-existing bilateral nephrostomy tubes while standard retrograde irrigation was initially maintained. After continuous antegrade saline flow across the stone was confirmed, retrograde irrigation was disconnected, and the ureteroscope irrigation channel was opened to allow passive outflow. The antegrade flow facilitated the distal migration of stone fragments, minimized proximal retropulsion during lithotripsy, and improved endoscopic visualization. Residual fragments were retrieved using a Dormia basket, followed by bilateral double-J ureteral stent placement.

The total operative time was two hours. The postoperative course was uneventful, with no infectious or surgical complications recorded according to the Clavien-Dindo classification. The nephrostomy tubes were removed on postoperative day 2, and the patient was discharged on postoperative day 3.

**Figure 7:**
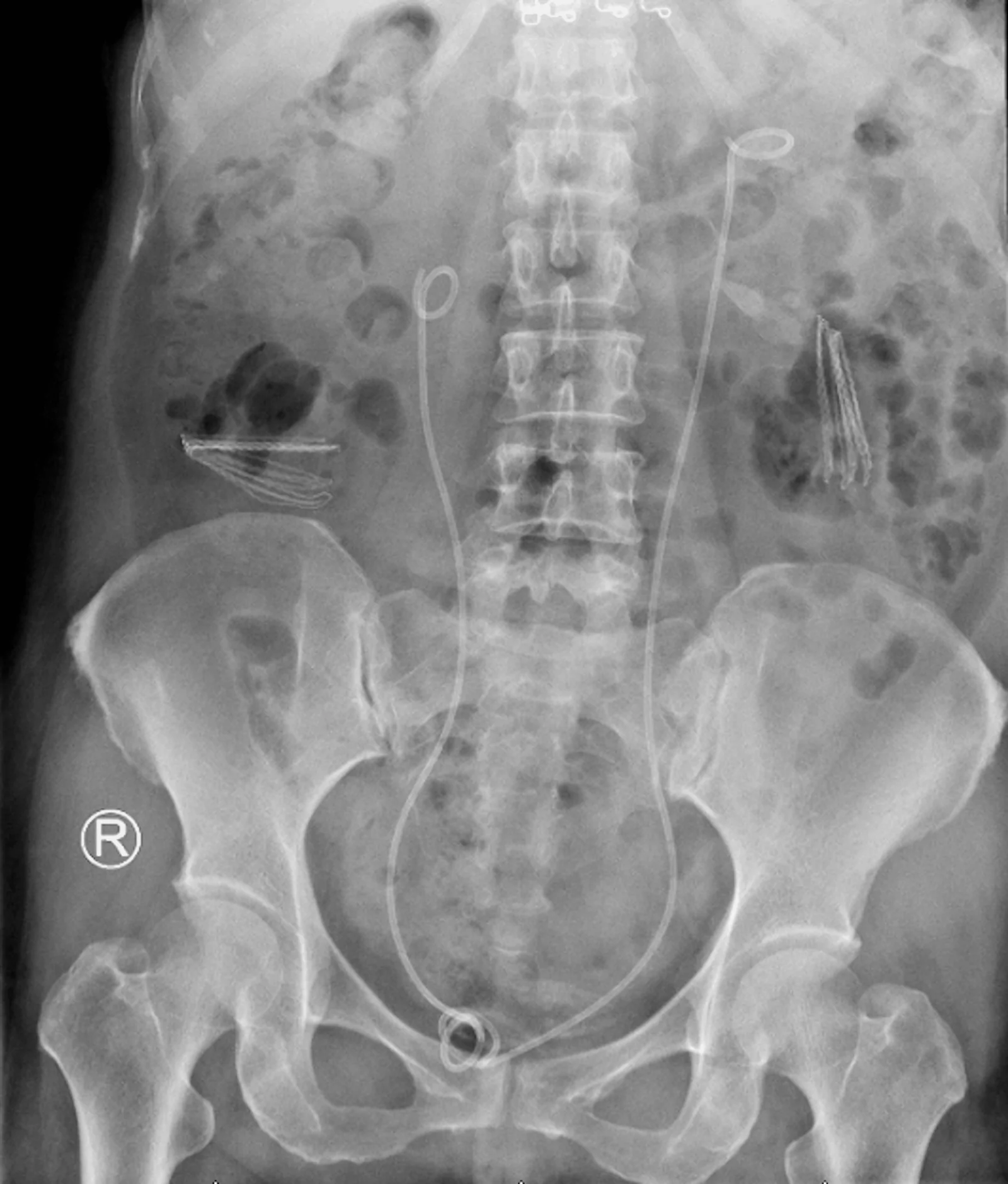
Postoperative KUB radiograph showing no visible residual stones in either ureter. KUB: Kidney-ureter-bladder.

Stone-free status was assessed using kidney-ureter-bladder (KUB) radiography performed one week postoperatively, which demonstrated no visible residual ureteral calculi (Figure 7). The ureteral stents were removed two weeks postoperatively. No stone composition analysis was performed.

## Discussion

Antegrade irrigation through a PCN tube during URS may represent a valuable adjunctive technique, particularly for optimizing stone-fragment clearance and minimizing retropulsion. In this case, we successfully applied continuous gravity-assisted antegrade irrigation through pre-existing bilateral PCN tubes during semirigid ureteroscopic laser lithotripsy for distal ureteral stones. The antegrade flow promoted the distal migration of fragments while reducing proximal retropulsion [4]. This facilitated efficient basket extraction and resulted in no visible residual ureteral stones on follow-up imaging after a single session.

The concept of antegrade irrigation is well supported in the literature for proximal or upper ureteral stones. Jung W et al. conducted a comparative study of 134 patients undergoing semirigid URS for upper ureteral stones, dividing them into antegrade irrigation through PCN and conventional retrograde irrigation groups. The antegrade group demonstrated a significantly higher stone-free rate (SFR) and shorter operative time without an increase in complications. Similarly, Kwon SY et al. reported superior outcomes in patients undergoing URS with PCN compared with those undergoing URS without PCN, including higher success rates and shorter operative times, with comparable safety profiles. However, within the PCN subgroup, external irrigation was associated with a numerically shorter operative time than no irrigation, but the difference was not statistically significant [1,4].

These benefits may result from several mechanistic advantages. Antegrade flow helps maintain a clear visual field by flushing debris distally, may decompress the collecting system, and creates a natural downstream current that counters the tendency of fragments to migrate proximally during laser fragmentation, a common issue with semirigid URS that can prolong procedures and increase the need for secondary interventions. Techniques such as Stone Cone devices, baskets, and gels have been described to mitigate retropulsion, but antegrade irrigation offers a simple, low-cost adjunct that utilizes existing drainage access [5].

Potential advantages in the distal setting include reduced intrarenal pressure buildup, a lower risk of fragment retropulsion into the kidney, particularly relevant in the presence of concurrent calyceal stones, as in our patient, and efficient fragment management in complex bilateral cases. No irrigation-related complications were observed, consistent with the literature demonstrating a preserved safety profile [4].

To our knowledge, reports specifically describing antegrade irrigation through pre-existing PCN tubes during semirigid URS for distal ureteral stones remain limited, making this case noteworthy. Most existing evidence focuses on proximal or impacted upper ureteral calculi, for which antegrade approaches, including combined antegrade-retrograde “rendezvous” techniques, are more commonly discussed [6]. Extending this technique to distal stones in patients with indwelling PCN tubes, which are often placed for initial obstruction and suspected infection, appeared feasible and effective in this case, with no visible residual ureteral stones on follow-up imaging.

Supporting literature includes Dellabella M et al., who described a high-pressure irrigation technique using an occlusive percutaneous balloon catheter for large, impacted proximal ureteral calculi, facilitating fragment dislocation and clearance [7]. Yoo J et al. further demonstrated successful antegrade irrigation-assisted ureteroscopic lithotripsy in patients with PCN tubes following urinary diversion for complicated UTIs [8]. More recently, Battikha A et al. evaluated retrograde pyeloperfusion during simulated percutaneous nephrolithotomy in silicone kidney and ureter models and demonstrated reduced antegrade ureteral fragment migration. Shalaby EA et al. evaluated forced diuresis as an anti-retropulsion technique during semirigid URS with pneumatic lithotripsy for lower ureteral stones. Although these techniques differ from gravity-assisted antegrade irrigation through PCN tubes, they support the broader principle of using controlled flow to improve fragment management and reduce retropulsion [9,10].

Limitations

This report has several limitations. First, it describes the experience of a single patient, limiting the generalizability of the findings and precluding conclusions regarding the efficacy or superiority of antegrade irrigation over conventional ureteroscopic techniques. Second, postoperative stone-free status was assessed using KUB radiography rather than NCCT, which is more sensitive for detecting small residual stone fragments. Third, several quantitative intraoperative parameters, including laser time, fluoroscopy time, irrigation pressure, and irrigation volume, were not routinely recorded and therefore could not be analyzed. In addition, stone-composition analysis and inflammatory markers such as CRP were unavailable. Despite these limitations, this case demonstrates the technical feasibility and successful application of continuous gravity-assisted antegrade irrigation through pre-existing PCN tubes during semirigid ureteroscopic laser lithotripsy for bilateral distal ureteral stones.

## Conclusions

Antegrade irrigation through pre-existing PCN tubes may serve as a simple and practical adjunct during semirigid ureteroscopic laser lithotripsy in selected patients with distal ureteral calculi. In this case, the technique facilitated stone-fragment management and retrieval while minimizing proximal retropulsion, with no visible residual ureteral stones on postoperative imaging and no postoperative complications. Further prospective studies are required to establish its safety, reproducibility, and clinical efficacy.
